# Integrating safer conception services into primary care: providers’ perspectives

**DOI:** 10.1186/s12889-019-6904-0

**Published:** 2019-05-09

**Authors:** Mariya C. Patwa, Jean Bassett, Leah Holmes, Lillian Mutunga, Mutsa Mudavanhu, Thembisile Makhomboti, Annelies Van Rie, Sheree R. Schwartz

**Affiliations:** 1 Johns Hopkins Bloomber School of Public Health, 615 N Wolfe St. E7136, Baltimore, MD USA; 2 Witkoppen Health and Welfare Centre, Johannesburg, South Africa; 3 Department of Epidemiology and Social Medicine, University of Antwerp, Antwerpen, Belgium

**Keywords:** Afer conception, Service integration, HIV, Family planning, HIV prevention

## Abstract

**Background:**

In 2012, South Africa adopted the Contraception and Fertility Planning guidelines to incorporate safer conception services into care for HIV-affected couples trying to conceive. These guidelines lacked clear implementation and training recommendations. The objective of this study was to investigate factors influencing integration of safer conception services in a clinical setting.

**Methods:**

Twenty in-depth interviews were conducted between October–November 2017 with providers and staff at Witkoppen Clinic in Johannesburg, where the *Sakh’umndeni* safer conception demonstration project had enrolled patients from July 2013–July 2017. Semi-structured interview guides engaged providers on their perspectives following the *Sakh’umndeni* project and possible integration plans to inform the translation of the stand-alone *Sakh’umndeni* services into a routine service. A grounded theory approach was used to code interviews and an adaptation of the Atun et al. (2010) ‘Integration of Targeted Interventions into Health Systems’ conceptual framework was applied as an analysis tool.

**Results:**

Five themes emerged: (1) The need for safer conception training; (2) The importance of messaging and demand generation; (3) A spectrum of views around the extent of integration of safer conception services; (4) Limitations of family planning services as an integration focal point; and (5) Benefits and challenges of a “couples-based” intervention. In-depth interviews suggested that counselors, as the first point of contact, should inform patients about safer conceptions services, followed by targeted reinforcement of safer conception messaging by all clinicians, and referral to more intensively trained safer conception providers.

**Conclusion:**

A safer conception counseling guide would facilitate consultations. While many providers felt that the services belonged in family planning, lack of HIV management skills, men and women trying to conceive within family planning may pose barriers.

## Background

People are living longer and healthier lives with HIV and many intend to have children [1–3]. For individuals and couples affected by HIV who are trying to become pregnant, safer conception has been promoted as an HIV combination prevention intervention to reduce HIV exposure to uninfected partners and prevent mother-to-child transmission at the very early stages of gestation. Safer conception strategies for resource limited settings include antiretroviral therapy (ART) and viral load monitoring for HIV-positive partners, HIV counseling and testing and initiation of pre-exposure prophylaxis for HIV negative partners, male medical circumcision, and education around risk reduction strategies, including self-insemination and timed condomless sex limited to the fertile window [4].

South Africa has the largest HIV epidemic globally, with an estimated 270,000 new HIV infections annually and 7.1 million people living with HIV [5]. Among people living with HIV, approximately 45% are estimated to be virally suppressed, and thus have a reduced risk of HIV transmission [5]. A core component of South Africa’s National Strategic Plan for HIV, TB and STIs is to strengthen integrated HIV services [6].

In 2011, the Southern African HIV Clinicians’ Society released guidelines for the inclusion of safer conception counseling into routine HIV care, which have recently been updated [4, 7]. However, these guidelines lacked clear implementation and training recommendations to incorporate safer conception services into facility operations [8]. The 2012 Department of Health (DOH) Contraception and Fertility Planning Guidelines recommended inclusion of safer conception services into antenatal, postnatal, and family planning spaces [9]. In 2017, the DOH “ideal clinic” standards promoted integrated HIV services with family planning and chronic care, suggesting that all clinicians in primary health care centers should be trained to provide safer conception services [10].

Despite a supportive policy environment, implementation of safer conception services has been limited to research demonstration projects, one of which is the *Sakh’umndeni* Safer Conception Clinic at the Witkoppen Health and Welfare Centre (Witkoppen Clinic) in northern Johannesburg [8, 11]. *Sakh’umndeni* enrolled 526 clients over a four-year period (2013–2017) [11–13]. In alignment with national guidelines, Witkoppen Clinic sought to translate its stand-alone safer conception services into a more scalable, integrated service delivery model using the lessons learned from *Sakh’umndeni*.

The objective of this study was to inform health systems scale-up of safer conception care by investigating factors that influence the integration, or ‘adoption system’, of safer conception services into a primary healthcare setting. We employed a modification of the ‘Integration of Targeted Interventions into Health Systems’ conceptual analysis framework to identify barriers to service integration, assess training needs, and examine provider buy-in and preferences for safer conception service delivery [14].

## Methods

### Study setting and study design

Witkoppen Clinic is a primary health care center and non-governmental organization based in northern Johannesburg, serving the surrounding areas of Fourways, Diepsloot, and informal settlement populations. Witkoppen provides comprehensive care including HIV and tuberculosis screening and treatment, family planning, antenatal and postnatal care, and mental health care.

A demonstration research study, evaluating the real-world effectiveness of safer conception interventions was implemented at Witkoppen Clinic from 2013 to 2017. Safer conception was provided as a stand-alone service, called the *Sakh’umndeni* Safer Conception Clinic – the development of which was informed by scientific literature as well as formative patient and provider interviews conducted in 2013 and previously reported [12, 15]. During the study period patients were recruited from the Witkoppen Clinic patient population and through outreach efforts within the catchment area of the clinic. Clinic providers and staff were encouraged to inform women and men in a relationship affected by HIV and desiring a baby of the *Sakh’umndeni* service and refer eligible individuals to the on-site service [11–13].

Upon completion of the demonstration project, in-depth interviews were conducted with healthcare providers as well as clinic and research staff to inform the health systems translation of the demonstration project into an integrated routine safer conception service at Witkoppen Clinic. Patient interviews, though previously included to design the service package and implementation, were not included at this stage of data collection.

### Data collection

Twenty in-depth interviews (IDIs) were conducted between October–November 2017 with Witkoppen Clinic healthcare providers and *Sakh’umndeni* research staff. Interviewees were purposively sampled using maximum variation to represent all healthcare provider cadres and staff providing HIV care, those knowledgeable about the clinic operations, and those with expertise in providing safer conception services. The interviews (three men and seventeen women) included three doctors, one senior manager, seven nurses, two auxiliary nurses, two researchers, and five HIV counselors.

Semi-structured interview guides engaged on provider perspectives and feedback on the original *Sakh’umndeni* safer conception services at Witkoppen Clinic, as well as safer conception integration plans. As staff positions interviewed and clinical providers at Witkoppen all speak English, interviews were conducted in English and averaged 20–30 min. Interviews were conducted, audio-recorded, and transcribed by MP.

All participants provided written informed consent. Ethical approval was obtained from the Human Research Ethics Committee at the University of Witwatersrand in Johannesburg, South Africa (protocol M130467).

### Analytic framework

**Fig. 1:**
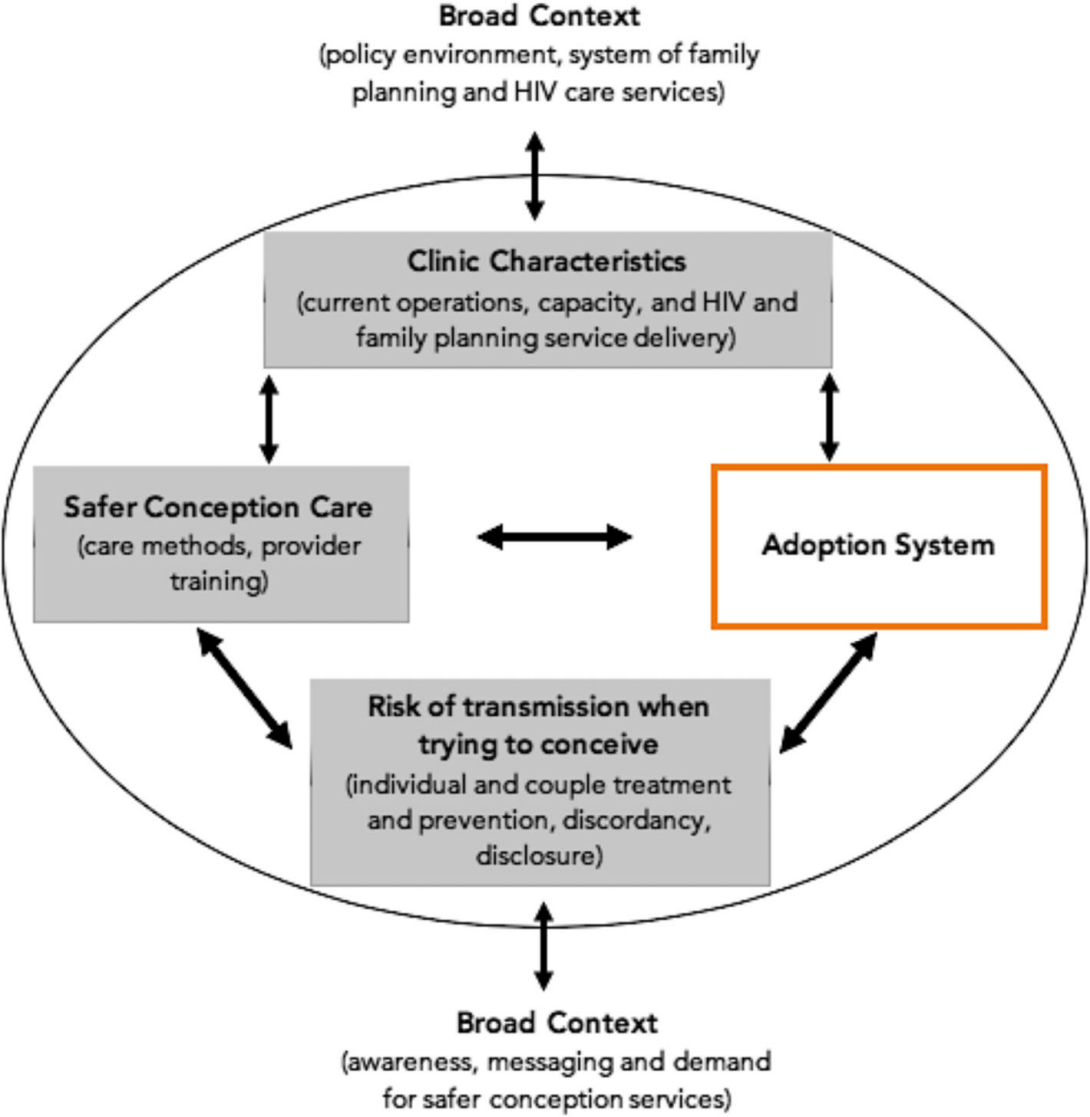
Adapted Atun et al. (2010) Framework

We adapted the ‘Integration of Targeted Interventions into Health Systems’ conceptual framework as an analysis tool (Fig. 1) [14]. This framework deconstructs factors associated with integration of targeted health services in order to capture the complexity of service integration and recognizes how different “adoption system” suggestions interact with important model features. Atun et al. (2010) identified healthcare providers as key actors or “adopters” who have an influence on the “adoption system” and subsequent delivery of services. In this application, the “problem” is the risk of HIV horizontal and vertical transmission – which will be referred to as “risk of transmission” – within families while trying to conceive and the “intervention” is safer conception care. The “clinic characteristics” refer to Witkoppen Clinic’s operations as an example of a busy (~ 8500 patient per month) urban primary care clinic in sub-Saharan Africa. The “broad context” is characterized by the generalized HIV epidemic within a policy environment that is resource limited, but supportive of the safer conception concept [14].

### Analysis

A grounded theory approach was used to understand provider perspectives on the integration or “adoption” of safer conception services, training needs, the link between family planning and safer conception, and outreach efforts [16]. Transcripts were independently analyzed and coded by MP and LH to identify patterns across interviews. The interview guide was used to develop initial codes. A codebook, consisting of 13 descriptive and interpretive codes was then developed, discussed, modified and agreed upon by MP and LH. Codes were then deductively grouped into themes relevant to safer conception integration. Analysis was completed using Atlas.ti 8 (*Berlin, Germany)*.

## Results

Overall, the safer conception demonstration project was well received by health care providers. Safer conception was unanimously perceived as a beneficial service for people living with HIV so that they can achieve their reproductive goals and improve their health outcomes. Future integration of safer conception into routine care at the clinic received the support of most health care workers.

*“Safer conception is a concept that has been in the facility for many years… For me I think it should actually be a culture. If we want to have zero new HIV infections by 2030 or 2020…according to the national strategic plan, we have to make sure that everybody takes responsibility of that.” (Manager)*

*“For me safer conception it was something good that happens for Witkoppen. I think it has touched a lot of people’s life… Because, really and truly when you really struggling to deal with the fact of you being diagnosed HIV positive, but still you really want to have this family, and at the end of the day you have your fears… if there is someone who is stepping in and saying it’s okay, you can still have a child, it’s okay, you are in safe hand, it’s okay we’ve got all the information that you need.*” *(Sakh’umndeni staff)*

Using the ‘Integration of Targeted Interventions into Health Systems’ adapted conceptual framework five themes emerged (Fig. 1) (1) the need for safer conception services training as a critical step to improve provider understanding of “safer conception care”; (2) messaging and demand generation to engage and improve the “broad context” for safer conception service awareness; (3) defining the extent of integration of safer conception services into routine care by recognizing and modifying “clinic characteristics” such as clinic operations and capacity for service delivery; (4) limitations of current family planning services as part of the “broad context” and “clinic characteristics”; (5) balancing the benefits of a “couples-based” intervention with the need of safer conception services for unaccompanied individuals and those who have not yet disclosed their HIV status.

### Need for safer conception training

Clinic doctors and some nurses were able to describe key service components but many reported discomfort providing safer conception services themselves without safer conception training. Self-insemination, in particular, was a method that providers voiced uncertainty about.

*“I try to share whatever information so that they conceive safely. I tell them about the viral load and when is the correct time to conceive like time, ovulation, and stuff like that… [The Sakh’umndeni research clinician] used to [teach] this artificial insemination and all those things I don’t know about. I don’t know how it’s done, but then I encourage them to get tested, both of them if the other partner is not tested yet to test, get their viral loads to low and undetectable before they try for a baby.” (Nurse)*

Other clinicians and counselors reported a general understanding of the purpose of the services, but relied on the safer conception clinic and dedicated service providers, suggesting a limited understanding of how to provide safer conception counseling.

*“They were helping positive or positive patients to really conceive… well guiding them with the whole transition of how to ensure that the partner remains negative if it was a discordant couple… I didn’t know much into the details, but I knew it took a lot of time with the consultations…and that [the Sakh’umndeni research clinician] was very dedicated.” (Nurse)*

*“The only thing that I understand about safer is… it helps them to continue having their status if they are negative then you are having a positive partner… Yeah, I know the only thing that I understand is that it’s for couples they are trying to do a baby whilst someone is positive and the other is negative or both positive.” (Counselor)*

Providers were open to receiving technical training in safer conception counseling, and also noted areas outside of safer conception methods that should be emphasized for all cadres of staff.

*“I think the first thing is [provider and staff] understanding of HIV. And, the importance of virologic suppression and not infecting either partner. Or transmitting the virus through pregnancy... Just the physiology of it.” (Doctor)*

Counselors were often referenced as a critical point of contact for patients, making it essential to build their skills, particularly in education around conception methods, including timing of intercourse and self-insemination, as well as to navigate relationship dynamics and ethics:

*“I would love to have training upon [safer conception]… one thing that I’m scared of, I don’t want to give information that I’m not sure about… I need to be more specific… for instance, in safer sometime self-insemination using syringes and all that, people [might] not feel comfortable with that. So, if they can ask me and say, ‘what’s the next option?’” (Counselor)*

*“Where do you begin? Okay the history taking and then the element of working around the menstrual cycle, how do you calculate that? Which [insemination method] is more efficient, which one would suit which couple…to know when to use which method? … How do you refer to other departments if there is a psychologist that needs to be included… what happens if you come to the realization that this couple they want to conceive, but financially does not make it right? How do you incorporate all elements to ensure that this is the best suitable thing for this couple, you know? … If you get couples who have 5 kids already, now they want to do it the right method…how do you incorporate that?…What governs me as a clinician to say this is the correct thing for you…” (Nurse)*

### Messaging and demand generation

When asked to identify activities that could facilitate service promotion and engage clients who would benefit from the service, providers wanted more explicit messaging about what the service is and how it can help their patients.

*“Maybe the meaning of safer conception - what does it mean?... Who also qualifies for being in the safer conception… what are the things that you need to tell this patient who is HIV negative or this patient who doesn’t know the partner status or this patient who want to have a family? What is your goal?” (Counselor)*

Informants suggested the development of posters, flyers and pamphlets in order to elicit patient awareness and questions about the service:

*“I would put posters up, you know, ‘are you thinking about falling pregnant?’ Maybe some patients sit for so long, I don’t know how much they look at posters, but probably ones that have more graphics… you know the pictures… ‘do you know it’s possible to have a negative baby? Do you know how to have a negative baby?’ Which could possibly prompt them to ask the question if perhaps the clinician doesn’t.” (Doctor)*

*“You give talks and if you have pamphlets just give them pamphlets because sometimes we have patients who are sick here so maybe that time [they are] not even listening to you, so if you give the pamphlet…when [they] get home can just read it.” (Counselor)*

Several providers and counselors found it critical to share this information outside of the clinic to reach people who are less likely to be aware of safer conception services, whom also have great need:

*“Clinic wise I think we are limiting people… I think there’s a lot of people out there who need the very same knowledge that we have and the very same knowledge that our patients will be receiving at Witkoppen. If we really want to be a good health facility, I think spreading the word broadly would actually, I don’t know, it would be a good thing for people to find out. Not a lot of people know about these things, you know. They still have that mentality that when you’re positive you know it’s like life has ended for you.” (Nurse)*

As a service that aims to prevent and treat HIV among both men and women planning to expand their families, it was important to gain perspectives on male engagement. Healthcare providers believed that men have an interest in safer conception services, thus it may be a way to reach men, but stressed the need for confidentiality of the services. Many suggested opportunities for synergies between the male-friendly service at Witkoppen Clinic and safer conception services.

*“…when you talk about kids, when a man wants a child he can stand up and go to the clinic. I’ve seen them coming… like doing follow-ups without their partners on safer conception [research study]. I think men will do.” (Sakh’umndeni staff)*

*“They don’t want to be tested HIV… they don’t want to use condom… I think the only thing we got a men’s clinic…But I know that, you know, when men want to have babies they can do everything, anything… I feel that they will use the service… maybe once or twice, but not always. Not always, but because of the male-friendly service and if we can work together maybe we can.” (Nurse)*

*“I mean the one is the men’s clinic. If we think that’s a priority and to specifically talk to just stay aware of discussing fertility plans with men as well as with women. Cause I don’t think, I may be wrong, but I certainly never have. I routinely ask the mothers, I’ve never asked our fathers if they are planning.” (Doctor)*

*“I think they would engage themselves in this and I believe with men you need to give them the element of trust that whatever that we saw here will not leak anywhere else. I think confidentiality is the only thing that actually makes men able to come in and use the services.” (Nurse)*

### Extent of integration of safer conception services

The implementation of safer conception services could follow different approaches. Some health care workers supported provision of safer conception services by all health care workers to make visits more convenient and efficient for patients.

*“I would wish that all the clinicians would know how to do this. So that like, if we talk integration, we are talking if for example a patient came to me I treat for pneumonia, if they want the safer conception I do it in the same consultation instead of having to send the patient somewhere. So, I think like it starts with the screening and then we start with what needs to be done after screening for convenience of the patient and it’s quite easier for the patient to get all the services in one consulting room.” (Nurse)*

Most providers and staff supported the introduction of safer conception services by counselors as the first point of contact with patients, followed by reinforcing safer conception messages by clinicians, and a strong referral system to more intensively trained safer conception providers.

*“If it’s a first timer, they will definitely have to come through the lay counselors for health talks. So, everybody does come through that when they come to the facility. And they’re the best place to give that information… so that I think that would really be the starting point.” (Manager)*

*“If they can go around like early in the morning in health talks…obviously [we counselors] can’t just go all over the process like in detail, but that little information that we’ll be dropping then they can ask to another clinician so that the patient can have more information.” (Counselor)*

A preference for partial integration was often based on concerns around long consultation times and the need to discuss sensitive issues.

*“Firstly, you need time, you [need] to have space to do a proper consultation. And really explain… all the different facets in terms of the virus, in terms of how to fall pregnant, and follow-up afterwards with the pregnancy and the baby. You need to have enough time, I believe that consistency in clinician is important.” (Doctor)*

*“Although I understand the rationale behind saying all services must be integrated… If you integrated it totally, so that everybody gets to do it, then you’re going to land up seeing somebody different every time… They’re not easy conversations to have with somebody… partners talking about what’s going on at home and their sex lives and everything else is not the sort of thing that you then want to start all over again the following month and tell somebody else all this stuff all over again and so I think the continuity [is important]. I think there’s a place for specialist care. I have reservations about every clinician doing everything, because I think it’s very difficult to maintain excellent quality of care with every clinician doing it.” (Doctor)*

Providers considered integration and more intensive training of clinicians in the antenatal and postnatal space.

*“I think I would probably train… in the antenatal [and] postnatal department [so] that you can have a couple of people but not just one clinician, because hopefully the patient will fall pregnant and then stay in the same environment. So, your counselors will be more likely to be similar, that’s as far as you can integrate it. It may not be one specific person but in that department [there’s] just more awareness of pregnancy and also as you go forward they’ll be staying in the same place.” (Doctor)*

### Current limitations of family planning services

The current South African Contraception and Fertility guidelines suggest the integration of safer conception services within family planning. Many health care workers reported that people perceive family planning as a service for prevention of pregnancy, not overall reproductive counseling. Patients who are being referred to family planning services are patients who do not want to conceive:

*“…if I wasn’t a clinician as well, from growing up all I know is that family planning is all about preventing a baby so… from home that’s what we are being taught you need to go to the clinic for prevention [of pregnancy], not necessarily you know for you to plan and prepare… it is a problem, because if maybe we patients, maybe the community as a whole we can view it in that way that even if for preparation…we grow up with, it’s all about [pregnancy] prevention but not necessarily about planning.” (Nurse)*

Clinicians generally agreed that the family planning culture among healthcare providers does not include the concept of planning and preparing for pregnancy.

*“I mean certainly my impression here is that our family planning clinic…is totally about [pregnancy] prevention…it doesn’t come across as a space where there’s enormous kind of innovation and enthusiasm for something new it seems to be something just ‘this is what we do and we dish out injections and we dish out pills’.” (Doctor)*

One clinician considered training family planning providers to present more options to patients, but placed the final responsibility on HIV clinicians to discuss patients’ fertility plans as family planning providers are not trained in HIV treatment initiation and management, posing a challenge to provision of safer conception care within family planning services [17].

*“I certainly think it would be beneficial to the patients if one can train your provider in family planning to have the broader discussion…even in the rest of the clinic and particularly HIV clinicians should try and have the discussion… ‘you know, you think about if you do want to fall pregnant, you know, speak to us first’…” (Doctor)*

### Benefits and challenges of a “couples-based” intervention

Several clinicians highlighted the value of seeing patients as a couple, emphasizing the importance of explaining key concepts of safer conception methods and HIV prevention and treatment to both partners:

*“…it’s always best to get the same information at the same time as a couple. Because like if I come here and you tell me something and you go and tell my partner I might distort the message, but if we hear the same message at the same time it’s actually easier for the couple.” (Nurse)*

*“I prefer seeing them as a couple because I’m more able to educate them with regards to conceiving safely.” (Nurse)*

However, provider preference towards seeing couples together poses the risk of excluding unaccompanied individuals from receiving information and possible prevention benefits associated with safer conception services. The issue of disclosure between partners was often cited as a critical barrier to the success and reach of safer conception services and HIV prevention efforts more broadly.

*“You know you should remember we are dealing with people who others they haven’t disclosed… others they did disclose… some they haven’t disclosed to their families. Those are the ones who are affected. So, it’s still an issue but what we encourage…to make sure that they understand and see the obstacle or the challenge that they will encounter in future if not disclosing.” (Counselor)*

## Discussion

Employing the ‘Integration of Targeted Intervention Health Interventions’ adapted framework (Fig. 1), our results suggest that most health care providers at Witkoppen Clinic were supportive of safer conception services and their integration into routine clinic care. Healthcare providers supported an implementation model where fertility conversations with HIV affected patients are initiated and safer conception services are introduced by counselors at the first point of contact. Subsequently, all clinicians could reinforce safer conception messages and refer eligible couples and women to a subset of more intensively trained, safer conception providers in the antenatal and postnatal care space. Our results suggest that integration of safer conception into family planning may be difficult, primarily due to the perception and structure of family planning as a pregnancy prevention focused service. Successful integration of safer conception into routine primary care would require community sensitization campaigns to educate the community and generate further demand for the services, safer conception training to improve provider understanding of safer conception care, and would need to balance the benefits of a “couples-based” intervention with the need for safer conception services for unaccompanied individuals and those who have not yet disclosed their HIV status. Given that few safer conception services have been operated in sub-Saharan Africa, provider feedback and buy-in from this setting is uniquely placed to translate the *Sakh’umndeni* demonstration project into routine practice.

In South Africa, Department of Health guidelines suggest integration of safer conception services into family planning spaces [9]. Our results show that providers and patients associate family planning services with pregnancy prevention, not a reproductive counseling space, echoing the concerns raised by Mmeje et al. (2015) [18]. Integrating safer conception within family planning may thus limit rather than facilitate access to and awareness of safer conception services. It may also exclude men from conversations on reproductive planning. Furthermore, family planning providers are often not trained to manage HIV treatment [8, 9]. Efforts made to test the feasibility and effectiveness of integrating safer conception care into existing family planning services may further elucidate training needs of family planning care providers and potential barriers to client uptake prior to scale-up, though these results suggest other models should also be tested [19]. Antenatal and postnatal care were identified as alternative services to incorporate safer conception services at the Witkoppen Clinic, so that women can transition from postnatal care into safer conception services and from conception services into antenatal care. These providers similarly have strong reproductive health backgrounds and may be better equipped to counsel and support patients with core safer conception methods as they are already trained to provide HIV treatment and fully understand the importance of viral suppression [10]. While this alternative integration model has advantages, men’s engagement in antenatal care has historically been minimal in South Africa and demand generation external to these services would be essential as pregnant and recently postpartum women are not the individuals most likely to require immediate safer conception care [20, 21].

Our results highlight that a supportive policy environment needs to be accompanied by training to successfully translate policy into practice. While providers were knowledgeable about safer conception service methods in our study setting, they expressed reluctance to engage discussions with patients on safer conception topics without concrete and specified training. This observation is similar to that of a study in Uganda which highlighted provider interest in and knowledge of safer conception service delivery, but a lack of implementation and training guidelines were considered a barrier to implementation [22]. A safer conception “toolkit”, including a training package and counseling guide should be created for training and implementation of safer conception services on a larger scale [12, 13, 23, 24]. Training must cover the available safer conception methods, emphasize counseling methods for lay counselors and providers, and include a guide for service providers to refer to in order to address common questions and concerns of clients. Ideally, following training, providers would be able to complete a full sexual and reproductive health assessment of both male and female partners, counsel on safer conception options, ensure viral suppression of HIV-positive partners, review menstrual diaries, and address patient concerns about chosen methods [3]. Training should not only cover guidelines and safer conception methods, but also focus on training of health care workers on how to communicate sensitive issues related to safer conception.

Time constraints and the need to continue safer conception care over a period of time were identified as important challenges to high quality safer conception service delivery. While providers were convinced of the value of promoting safer conception, many expressed concerns with the length of time required per consultation and the number of patients they were expected to counsel with limited time and staff. Switching between care providers at different safer conception follow-up visits, which would occur when transitioning from a stand-alone service to integrated care, was raised as an important concern given the intimate nature of information shared within safer conception discussions [4, 7]. We suggest inclusion of a condensed safer conception checklist or form to include into patient files for providers to minimize the number of times sensitive questions are raised by providers.

In our study, providers voiced a preference for working with couples, which presents benefits and potential challenges. In Kenya, a safer conception demonstration project showed that a couples-based approach improved tailored counseling to couples’ preferences [25]. Similarly, in prevention of mother-to-child HIV transmission studies, male partner involvement shows an association with increased and effective uptake of interventions – some of which overlap with safer conception methods [4, 7, 26]. The WHO provides recommendations for couples-based counseling to support disclosure of HIV status [27]. However, while safer conception may present the opportunity for couple engagement and male partner involvement, it should not preclude services to women (or men) who present unaccompanied or have not yet disclosed their HIV status. This challenge is real and can undermine HIV transmission efforts, as shown by denial of services to sex workers, who were turned away from antenatal services promoting male involvement when unaccompanied by a partner [28].

This study has several limitations. First, the study was conducted at a single primary care clinic and thus certain results may not be generalizable. However, utilization of the ‘Integration of Targeted Interventions into Health Systems’ conceptual framework is intended to further contextualize the results and promote their relatability on a larger scale. Furthermore, given that there have been very few safer conception services implemented in primary health settings in Sub-Saharan Africa, a key strength of this study is that it is based at one of the only sites that has implemented this work and thus lessons around exposure and integration of services are not merely hypothetical but based on grounded experiences which add a unique value that most other locations could not address. Furthermore, the safer conception package evaluated is based on national and international guidelines, and the barriers to service implementation that were highlighted, including disclosure between partners, time and staff constraints, and limitations of family planning services are common to most sub-Saharan African primary health care facilities [23, 29–31].

As care providers and staff interviewed at this site were already familiar with safer conception services through experiences with the stand-alone safer conception demonstration project at the clinic, it should be noted that barriers to integration are likely even greater in other settings depending on the baseline safer conception knowledge of providers, capacity for implementation, and collection of reportable indicators. Purposive sampling was used to ensure a diversity of provider type experiences were represented. However, response bias was considered - providers may have been more willing to share positive perspectives of safer conception service delivery if they had positive relationships with members of the safer conception demonstration project. The research team was cognizant of the importance of highlighting responses of the actual participants, ensuring that their voices were heard and that discrepant viewpoints were documented. Additionally, in the current study, only provider interviews were conducted at the site to inform a modified "adoption system" of safer conception services. However, patient interviews have been previously conducted at this site to establish a need for safer conception services and inform the design of the *Sakh’umndeni* clinic [12, 15]. For the purposes of this study, provider feedback and buy-in was critical to support the transition of the safer conception research effort to implementation of safer conception services into routine practice.

Moving forward, client uptake and perceptions of integrated models of safer conception care will be critical to assess and improve routine safer conception practice. From the national to the provider to the household level, greater awareness of safer conception strategies and reproductive rights will be necessary to promote HIV prevention between couples trying to conceive and to future children.

## Conclusion

Provider perspectives on service delivery informed the “adoption system” for implementation and scale-up. A safer conception service model partially integrated within primary care, where counselors inform clients of safer conception services at the first point of contact within the clinic, followed by reinforcement of safer conception messaging by all clinicians, and referral of eligible individuals to more intensively trained safer conception providers was identified as the preferential model. Successful integration of safer conception services into routine HIV care will require community sensitization and provider training that pays attention to the technical aspects and the sensitive nature of safer conception counseling.

## Abbreviations

ART: Antiretroviral Therapy
DOH: Department of Health
IDIs: In-depth interviews
WHO: World Health Organization
Witkoppen Clinic: Witkoppen Health and Welfare Centre
